# Utility of Real-Time Quantitative Polymerase Chain Reaction in Detecting* Mycobacterium tuberculosis*

**DOI:** 10.1155/2017/1058579

**Published:** 2017-01-11

**Authors:** Zhongquan Lv, Mingxin Zhang, Hui Zhang, Xinxin Lu

**Affiliations:** ^1^Clinical Laboratory, Beijing Tongren Hospital, Capital Medical University, Beijing 100069, China; ^2^Tuberculosis Control Center, Chinese Center for Disease Control and Prevention, Beijing, China

## Abstract

This study aimed to assess the value of real-time quantitative polymerase chain reaction (RT-qPCR) for the detection of* Mycobacterium tuberculosis* (MTB). Samples from 192 patients with suspected MTB were examined by RT-qPCR and an improved Löwenstein–Jensen (L-J) culture method. To evaluate the diagnostic usefulness of RT-qPCR in detecting MTB, a receiver operating characteristic (ROC) curve for RT-qPCR was generated, and the area under the curve (AUC) as well as a cutoff value was calculated. Using the L-J culture method as the gold standard, accuracy of the RT-qPCR method for detecting MTB was 92.7%, with sensitivity and specificity of 62.5% and 97.02%, respectively. In comparison with the improved L-J culture method, the AUC of RT-qPCR ROC curve was 0.957, which was statistically significant (*p* < 0.001). The Youden Index reached the maximum value (0.88) for gene copy number of 794.5 IU/mL, which was used as the cutoff value. RT-qPCR detection of MTB yielded results consistent with those of the improved L-J culture method, with high accuracy. RT-qPCR may be used as an auxiliary method for etiological diagnosis of tuberculosis.

## 1. Introduction

Tuberculosis is an infectious disease which is seriously harmful to human health and is also one of the major public health concerns worldwide. According to the report of World Health Organization (WHO) in 2013, nearly 8.6 million of new cases of tuberculosis were reported in the world in 2012, with an incidence rate of about 122/100,000 and 1.3 million total deaths [1]. China is one of the 22 countries with a high burden of tuberculosis, accounting for 15% of the total global burden [2]. Tuberculosis prevalence in China is second only to that of India. Annual incidence of tuberculosis in China is estimated at 0.9–1.1 million, accounting for 12% of global incidence [1]. Meanwhile, China is also one of the 27 countries with high prevalence of multidrug resistant tuberculosis. Nearly 50% of global drug resistant tuberculosis cases are found in China and India [3].

Diagnosis of* Mycobacterium tuberculosis* (MTB) infection is made by acid-fast bacilli staining and the improved Löwenstein–Jensen (L-J) culture method. Acid-fast staining of the sputum smear is considered the gold standard diagnostic method used to confirm MTB infection worldwide. However, this method has several limitations. First, an important number of bacteria must be present in the sputum to yield an accurate reading, indicating its low sensitivity [4]. In addition, although acid-fast staining requires the integrity of the mycobacterial cell wall, bacterial viability is not necessary; for instance, it is difficult to distinguish between natural infection and Bacille Calmette-Guerin (BCG) immune infection by this method [5]. This indicates its low specificity. Furthermore, this method largely depends on laboratory conditions and the technological expertise of the personnel and is time-consuming [6]. While* M. tuberculosis* can hardly be stained by acid-fast dyes once in cells, extrapulmonary TB diagnosis is difficult, since the irregularly distributed bacilli tend to form clumps, which may show false negative results [7].

The improved L-J culture method has slightly higher sensitivity and specificity than the smear method. It is capable of distinguishing between dead and live bacteria, determining drug sensitivity. However, it is time-consuming, with the results usually available in 6–8 weeks. Non-MTB strains can also grow in the culture medium; therefore, it is necessary to perform the strain identification test to determine whether the strain growing is MTB or not. Therefore, the improved L-J culture method might not totally meet the requirements for clinical application.

In 1989, Hance et al. [8] firstly applied PCR for the detection of MTB. PCR has high sensitivity and specificity and is capable of detecting 1–100 fg of purified MTB DNA [9]. The conventional PCR technique is hardly effective because of false positives from amplified “contaminant” DNA. Real-time quantitative PCR (RT-qPCR) is the most commonly used quantitative PCR method, in which the PCR reaction is prepared with the addition of a specific fluorescent dye. Since its introduction in 1996, RT-qPCR has played a vital role in basic and applied research in life sciences [10, 11].

Recent studies demonstrated that RT-qPCR is widely used in molecular diagnosis due to its specificity, high degree of automation, and good repeatability [12–14]. Marín et al. [15] detected multiple Rifampin and Isoniazid resistance mutations in MTB from respiratory samples using RT-PCR, revealing the minimum sensitivity to be as low as 1 × 10^3^ CFU/mL. Shrestha et al. [16] accurately detected and distinguished between MTB and non-MTB strains by RT-qPCR controlling solution temperature, which improved method's specificity. Ravva and Stanker [17] performed the detection of MTB DNA by the SYBR Green and TaqMan PCR methods and found the minimum detectable concentration to be as low as 0.34 FG, indicating that both the probe and dye methods have high specificity and identical detection efficiency.

To overcome the inherent limitations of traditional detection methods, it is urgent to develop a rapid detection technology for MTB, with high sensitivity and specificity. RT-qPCR not only maintains the characteristics of PCR such as high sensitivity and rapid detection but also overcomes the shortcomings of conventional PCR, including false positives and the lack of quantifiable results. It is expected that RT-qPCR would be used as a new diagnostic tool for tuberculosis. The present study aimed to assess the usefulness of RT-qPCR in MTB detection and compare the differences between RT-qPCR and the improved L-J culture method in detecting tuberculosis in samples from patients with suspected tuberculosis.

## 2. Materials and Methods

### 2.1. Samples

A total of 192 samples (from 118 males and 74 females) were collected from patients with suspected tuberculosis in each clinical department of the Beijing Tongren Hospital, Capital Medical University, from July 2006 to November 2014. The 192 samples included sputum from 107 cases, bronchoalveolar lavage fluid from 16 cases, urine from 36 cases, pleural effusion from 18 cases, cerebrospinal fluid from 8 cases, and pus from 7 cases. This study was approved by the ethics committee of Beijing Tongren Hospital, Capital Medical University (Beijing, China); written informed consent was obtained from every participant.

### 2.2. Methods

#### 2.2.1. Improved L-J Culture Method

The BACTEC-MGIT320* Mycobacterium* detection system and blood culture bottle were provided by Becton Dickinson (BD) (US). L-J culture medium, phosphate buffer solution (PBS, pH = 6.8), digestion solution, and other related reagents were prepared according to the “Laboratory Science Procedure of Diagnostic Bacteriology in Tuberculosis.” All procedures were strictly in compliance with the BACTEC-MGIT320 liquid culture method. For digestion-decontamination, equal volumes of specimen and the N-acetyl-l-cysteine- (NALC-) NaOH solution were mixed and vortexed for 20 minutes. The resulting mixture was transferred into a conical tube (less than 10 mL), with PBS (pH = 6.8) added immediately (less than 45 mL) before centrifugation for 15 minutes (3500 ×g). The supernatant was removed, and 1-2 mL PBS (pH = 6.8) was added to resuspend the precipitate, of which 0.5 mL was transferred to the BD blood culture bottle and cultured in the BACTEC-MGIT320 instrument after mixing. The results were evaluated according to the “Laboratory Science Procedure of Diagnostic Bacteriology in Tuberculosis.”

#### 2.2.2. DNA Isolation

DNA was extracted using DNA isolation solution from Shenzhen PG Biological Engineering Co., Ltd. (China), according to the manufacturer's instructions. Briefly, 2-3 volumes of 4% NaOH were added to sputum samples, mixed well, and shaken for 30 min at 37°C. Then, 0.9 mL of the liquefied sample was transferred into a 1.5 mL sterile centrifuge tube and centrifuged at 12500 ×g for 10 minutes, and the supernatant was removed. 1 mL of sterile saline was added to the precipitate, mixed, and vortexed. The samples were then centrifuged at 12500 ×g for 10 minutes, with the supernatant removed. The remaining body fluid samples were centrifuged at 12500 ×g for 10 minutes, with the supernatant discarded. After addition of DNA isolation solution (30 *μ*L) to the precipitate, centrifugation was carried out at 2000 ×g for 5 seconds. The sample was then incubated at 37°C for 30 minutes, followed by incubation at 100°C for 10 minutes and centrifugation at 12500 ×g for 10 minutes. A total of 2 *μ*L supernatant was used for the PCR reaction. To 40 *μ*L of negative and positive control standards, an equal volume of DNA extract was added, mixed well, and treated as described above.

#### 2.2.3. RT-qPCR

The MTB nucleic acid amplification (PCR) assay kit was purchased from Shenzhen PG Biological Engineering Co., Ltd. A total of 2 *μ*L DNA template was added to the PCR tube (containing 37.8 *μ*L PCR reaction mix, 0.2 *μ*L thermostable DNA polymerase, and four nucleotide monomers). The total reaction volume was 40 *μ*L. RT-qPCR was performed on an ABI 7300 fluorescent quantitative system (PE, USA) with amplification conditions as follows: 5 min incubation at 37°C; 1 min of initial denaturation at 94°C; and 40 cycles of 5 s at 95°C and 30 s at 60°C. TB-DNA amounts in the samples were calculated according to *C*_*t*_ values (gene copy number per mL). Positive criteria were typical S-shaped growth curve; the strongest fluorescence intensity was greater than basal fluorescence by 20 units, with the gene copy number above 1000 IU/mL.

#### 2.2.4. Statistical Analysis

Statistical analyses were performed using SPSS 11.5. Chi square test was performed for rate comparison between groups. To assess the diagnosis value of RT-qPCR in TB detection, the improved L-J culture method was selected as the gold standard. According to results obtained from both methods, receiver operating characteristic (ROC) curve analysis was generated, with area under the curve (AUC) of RT-qPCR calculated. The optimal diagnostic cutoff value was determined by calculating the Youden Index of the ROC curve. Statistical significance was set as *p* < 0.05.

## 3. Results

### 3.1. MTB Detection Using RT-qPCR Is Consistent with That of the Improved L-J Culture Method

Detection of MTB was performed in 192 samples using both RT-qPCR and the improved L-J culture method. Among the 192 samples, 15 cases were positive for MTB based on results in both methods; 5 cases were positive based on the improved L-J culture method but negative based on RT-qPCR results (false negatives); meanwhile, 9 cases were negative based on the improved L-J culture method but positive based on RT-qPCR results (false positives), and 163 cases were negative based on both methods (Table 1). Using the improved L-J culture method as the gold standard, the accuracy of MTB detection by RT-qPCR was determined to be 92.70%, with sensitivity and specificity of 62.50% and 97.02%, respectively.

One hundred and ninety-two samples from different tissues were analyzed using RT-qPCR and an improved L-J culture method (see Table 2). Of the 107 sputum samples, 17 tested positive for MTB by RT-qPCR (15.9%); 14 cases were screened out by the improved L-J culture method with a detection rate of 13.1%. Of the 16 alveolar lavage samples, two were positive in both RT-qPCR and the improved L-J culture method, while fluid samples showed a positive rate of 14.3%. In 36 urine samples, 2 cases were screened out by RT-qPCR (detection rate of 5.6%) and one by RT-qPCR (detection rate of 2.8%). Two cases were screened out by both RT-qPCR and the improved L-J culture method in 18 ascites and hydrothorax samples, with a positive rate of 11.1%. None of the 8 cerebrospinal fluid cases were screened out by either method, with a detection rate of 0%. One case was screened out by both RT-qPCR and the improved L-J culture method in 7 pus samples, with a positive rate of 14.3%. Differences in positive detection rates between RT-qPCR and the improved L-J culture method among samples from different tissues were not statistically significant (*p* > 0.05). These findings indicated that MTB detection using RT-qPCR yielded results consistent with those obtained with the improved L-J culture method.

### 3.2. RT-qPCR Is Highly Accurate for MTB Detection

**Figure 1:**
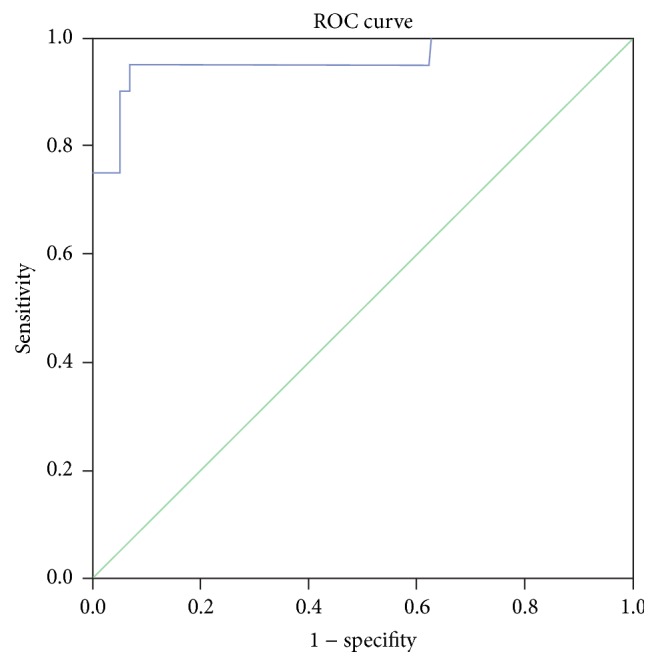
ROC curve of MTB DNA detection by RT-qPCR.

ROC curve analysis is widely applied as a statistical method in clinical diagnosis and population screening. Compared with the improved L-J culture method, the area under the ROC curve for RT-qPCR was 0.957 and it was statistically significant (*p* < 0.001) compared with the value of 0.5 which corresponds to the chance with no diagnostic value. These results demonstrated that RT-qPCR had a highly accurate rate of MTB detection (Figure 1).

By calculating the Youden Index for the ROC curve, we found that it reached the maximum value (0.88) with gene copy number of 794.5 IU/mL, which can be used as the optimal diagnostic cutoff value.

## 4. Discussion

Detection of MTB in samples from different tissues demonstrated that the RT-qPCR method has a higher positive detection rate compared with the traditional bacteriological detection method [18]. In the present study, by assessing MTB screening results in 192 samples from different tissues using the RT-qPCR and the improved L-J culture method, the overall positive detection rate of RT-qPCR method (12.5%) was higher than that of the improved L-J culture method (10.4%). In the remaining samples, the positive detection rate in sputum samples was higher in RT-qPCR (15.9%) data than those of the improved L-J culture method (13.1%); the positive detection rate by RT-qPCR (5.6%) in urine samples was also higher than that of the improved L-J culture method (2.8%). These results corroborated with previous studies. However, the correlation between RT-qPCR, which is considered a new molecular diagnostic method, and the conventional gold standard method (bacteriological detection) has not been reported. In the present study, 192 samples from different tissues were analyzed by RT-qPCR and the improved L-J culture method, and correlation between positive and negative detection rates by these two methods was analyzed. In comparison with the gold standard culture method, accuracy, sensitivity, and specificity of MTB detection using RT-qPCR were 92.70%, 62.50%, and 97.02%, respectively. Overall, there was high consistency between the two methods. Although the overall positive detection rate of RT-qPCR was higher than that of the traditional bacteriological detection method, the consistency of results still needs to be confirmed on a larger sample size. In clinical diagnosis of MTB, combination of RT-qPCR and bacteriological methods might improve diagnostic accuracy and save valuable time.

Sensitivity, specificity, and optimal clinical cutoff value for the RT-qPCR detection method of MTB have not been reported previously. The current study found the area under ROC curve of RT-qPCR to be 0.957 and it was statistically significant (*p* < 0.001). Thus, the RT-qPCR method has a diagnostic value as an indicator of tuberculosis. In addition, ROC curve analysis is helpful in determining the optimal clinical cutoff value. The closer the point on the curve is to the upper left-hand corner, the higher the accuracy of the test is. The point closest to the upper left-hand corner is the optimal clinical cutoff value with the lowest false positive and false negative rates.

Here we calculated the Youden Index of the ROC curve and found the maximum value (0.88) with gene copy number of 794.5 IU/mL, which was used as the optimal diagnostic cutoff value. In the present study, we first performed ROC curve analysis for RT-qPCR results, evaluated the reliability of RT-qPCR for tuberculosis diagnosis, and provided evidence for clinical utility of RT-qPCR in MTB detection by determining the optimal diagnostic cutoff value.

Laboratory detection of MTB is the foundation of tuberculosis diagnosis. Although the culture method is considered the gold standard, the growth cycle of MTB is long and results are usually returned in 1-2 months [19]. Therefore, the bacteriological detection method cannot fully meet the requirements of a clinical diagnostic tool. Compared with the traditional detection methods for pathogenic microorganisms, RT-qPCR is a closed amplification detection system, which detects specific DNA fragments of MTB. This method is highly sensitive and specific and convenient and rapid, with a high degree of automation.

## 5. Conclusions

RT-qPCR is especially suitable for identifying bacterial strains and drug resistant bacteria, which are difficult to culture or grow slowly. It remedies the shortcomings of traditional methods used to detect pathogenic bacteria, meets the requirements for early diagnosis of tuberculosis, and has good prospects for clinical application. Controlling the false positive and negative rates of RT-qPCR as well as standardizing, automating, and enhancing the method's repeatability and comparability is the focus of our future studies assessing RT-qPCR technology.

## Figures and Tables

**Table 1:** Comparison of MTB detection in 192 samples between RT-qPCR and the culture method.

Improved L-J medium culture	RT-qPCR		Total
	Positive	Negative	
Positive	15	5	20
Negative	9	163	172
Total	24	168	192

MTB, *Mycobacterium tuberculosis*.

L-J, Löwenstein–Jensen.

RT-qPCR, real-time quantitative PCR.

**Table 2:** Results of MTB detection by the two methods.

Sample source	Number	RT-qPCR		Improved L-J medium culture	
		Positive number	Positive rate (%)	Positive number	Positive rate (%)
Sputum	107	17	15.9	14	13.1
Alveolar lavage fluid	16	2	14.3	2	14.3
Urine	36	2	5.6	1	2.8
Ascites and hydrothorax	18	2	11.1	2	11.1
Cerebrospinal fluid	8	0	0.0	0	0.0
Pus	7	1	14.3	1	14.3
Total	192	24	12.5	20	10.4
